# Illuminating the human virome in health and disease

**DOI:** 10.1186/s13073-020-00766-x

**Published:** 2020-07-27

**Authors:** Fatemeh Adiliaghdam, Kate L. Jeffrey

**Affiliations:** 1 Department of Medicine, Division of Gastroenterology and the Center for the Study of Inflammatory Bowel Disease, Massachusetts General Hospital, Harvard Medical School, Boston, MA 02114 USA; 2 Center for Microbiome Informatics and Therapeutics, Massachusetts Institute of Technology, Cambridge, MA 02139 USA

## Abstract

Although the microbiome is established as an important regulator of health and disease, the role of viruses that inhabit asymptomatic humans (collectively, the virome) is less defined. While we are still characterizing what constitutes a healthy or diseased virome, an exciting next step is to move beyond correlations and toward identification of specific viruses and their precise mechanisms of beneficial or harmful immunomodulation. Illuminating this will represent a first step toward developing virome-focused therapies.

## Late to the party

In the study of microorganisms, bacteria frequently steal the limelight. During an influenza outbreak in late 1800, it was the bacterium *Haemophilus influenzae* isolated from sputum that was first presumed to cause disease. During the 1918 influenza pandemic, urgent efforts to isolate this causative bacterium failed and it was not until the 1930s that a filterable agent, a virus, *Influenza H1N1*, was identified as the culprit [1]. Similarly, in the pursuit of understanding human commensal microorganisms, the last 20 years of research has focused almost exclusively on bacteria and their regulation of our immune and nervous systems. In comparison, very little is known about eukaryotic and prokaryotic viruses that also inhabit asymptomatic humans. Given that the name *virus* was coined from the Latin word meaning *slimy liquid* or *poison* and that viruses are considered obligate pathogens, a possibly “beneficial virome” is surprising to many*.*

The late start for viruses in the commensal microorganism field is in large part due to our inability to readily culture or detect them, as was the case during the discovery of the influenza virus. We do not yet know the eukaryotic cell or bacterial host of most viruses, and there is no universal 16S ribosomal RNA equivalent, as in bacteria, allowing for rapid taxonomic characterization. Technologies such as metagenomics have only recently enabled identification of viruses in healthy human tissues. This initially involved sequencing all DNA or RNA in a sample (human, bacterial, and viral), and computationally aligning the massive number of sequences to identify those that resemble known viral genes. An improvement on this approach now involves filtering samples to purge eukaryotic cells and bacteria so that only virus-like particles (VLPs) remain for sequencing. However, since the virome consists of both temperate bacteriophages within bacterial genomes and free VLPs, both total and VLP sequencing will likely provide greater representation of all viruses. Nonetheless, with the approaches taken thus far, studies have revealed viruses are abundant in human feces, blood, skin, lung, oral cavity, and an array of other tissues of healthy and diseased individuals [2–5].

## A moving target

The human intestinal virome established at birth is dominated by bacteria-infecting viruses, while eukaryotic viruses gradually emerge after birth [6]. One gram of human feces contains around 10^8^–10^9^ VLPs, and exploratory sequencing has shown that the identifiable fraction of this virome is primarily bacteriophages, including dsDNA *Caudovirales,* ssDNA *Microviridae* [2], and the recently identified predominant CrAssphage [7]. Viruses that infect eukaryotic cells within the human fecal virome have been identified to belong to families *Astroviridae*, *Anelloviridae*, *Picornaviridae*, *Caliciviridae*, and *Herpesviridae*, among others [2, 3].

However, many hurdles in our ability to catalog the human virome remain, making this data far from final. (1) The vast majority of viruses share little to no homology with annotated viruses in reference databases. Viruses infecting animals, plants, fungi, and protozoans (collectively eukaryotic viruses) number around 100 million species while those infecting bacteria are estimated at 10 trillion, yet a large proportion remain unannotated. While the NCBI Virus Portal (https://www.ncbi.nlm.nih.gov/labs/virus/vssi/#/) is reporting new annotations at an exponential rate, virologists have struggled to distinguish clear classes and kingdoms for most of the virosphere, with the exception of a recently published taxonomic hierarchy [8]. Thus, reanalysis of relatively recent human virome publications may already be warranted. (2) Computational analysis methods vary considerably across virome studies, as this is a nascent field, making direct comparisons difficult. (3) Viruses rely on the host organism for successful replication; therefore, the discovery of viruses that specifically infect human cells may be better achieved by analysis of less accessible tissue and cells, rather than feces or bodily fluids, where they are likely scarce. (4) False positives in sequencing data remain an issue as many sequencing reagents, or DNA spike-ins during sequencing, are derived from bacteriophages or bacteria carrying bacteriophages. A consensus on laboratory techniques and computational analysis pipelines is a much-needed advance in the virome field. (5) We still have a very limited perspective on the healthy human tissue-specific virome. We know little about the virome in individuals from different geographic locations, in those consuming different diets, and in old or young individuals, and thus, it is difficult to discern cases of vertical and horizontal transmission or composition changes before, during, and after disease onset. A tighter grasp on what a healthy virome looks like—an equivalent to the Human Microbiome Project—would allow clearer inferences about how the virome influences disease.

## Making the leap from correlation to causation

Despite the present limitations in characterizing the human virome in health, robust fluctuations in the virome in multiple diseases have been reported. In inflammatory bowel disease (IBD), it was found that enteric *Caudovirales* temperate phage expanded, although the degree that this was due to alterations in bacteria remains unresolved [2, 3]. In colorectal cancer, virome signatures were shown to differentiate individuals at the early versus late stages of disease. In type I diabetes, expanded enteric bacteriophage diversity was found to precede disease and *Circoviridae* eukaryotic viruses were enriched in controls. In cystic fibrosis, sputum phage communities were highly similar and eukaryotic viral communities were found to be dominated by herpesviruses and retroviruses. In graft-versus-host disease (GVHD), a progressive expansion of eukaryotic gut viruses was shown to follow hematopoietic stem cell transplant, and picobirnaviruses were associated with early post-transplant GVHD. In HIV patients, low peripheral CD4 T cell counts were associated with an expansion of enteric adenoviruses [5]. However, this cataloging of diseased viromes is vastly outpacing our mechanistic understanding as we still do not know whether these altered viromes actually contribute to disease.

A lesson from the microbiome field, at this juncture in virome research, would be to move beyond correlations and toward a detailed analysis of how certain viruses autonomously or cooperatively educate our physiology. Functional studies in mice have found that enteric viruses inhabiting a healthy host provide immune and gut homeostasis. Depletion of viruses or virus receptors in healthy mice exacerbates intestinal inflammation while treatment with viral ligands protects from disease [5]. However, precise mechanisms by which individual viruses provide protection are limited. Furthermore, how human virome composition impacts health or disease remains ambiguous as direct functional studies are currently lacking. However, both prokaryotic and eukaryotic viromes possess the ability to directly immunomodulate their human hosts based on reports of trans-kingdom interactions of bacteriophage and human immune cells [9]. Furthermore, given that viruses exist in complex communities comprising bacteria, fungi, and protozoans, indirect outcomes of virome changes will almost definitely occur through alteration of surrounding microorganism communities. Another question and appealing avenue of investigation is whether commensal viruses impact the host’s ability to fight pathogenic viruses through tonic stimulation of antiviral immunity or if conversely, acute virus infection impacts the resident virome. Finally, since complex disease phenotypes are the result of environmental triggers in the context of genetic susceptibility, variable impact of the virome will depend on host genetics and should be considered. For instance, a loss-of-function variant of host virus receptor MDA-5 (encoded by gene *IFIH1*) associates with incidence of IBD but protects from type I diabetes [5] suggesting divergent roles for viruses in different contexts.

## Translating to diagnosis and therapy

The ultimate goal of virome research is to translate findings into diagnostic and therapeutic opportunities. With accurate mapping of the virome in different human tissues in healthy and disease states, we can begin to use certain viruses as biomarkers or attempt to manipulate virome signatures. Moving from association to causation will confidently enable us to harness the healthy virome or disrupt the disease-associated one. Uncoupling the roles of eukaryotic and prokaryotic viruses on immune state and improvements on propagation of individual candidate viruses for functional studies will advance these goals. Therapeutic avenues could also focus on the beneficial or detrimental host immune responses to viruses, rather than the viruses themselves to mitigate virome-related diseases. Viruses may also serve an important role in fecal microbiota transplants (FMTs) since filtered feces (removing the bacterial component) have the same efficacy in treating the *Clostridium difficile* patients [10]. However, there is still no knowledge of the virome composition of FMTs and no consensus on if this is something we should be measuring in donor and recipient patients. Finally, benign viruses within the healthy virome could conceivably be used for safe gene delivery into humans.

We have just begun to reveal the complexities and promise of the virome using computational genomics, but application of the virome remains relatively underexplored. Many challenges in the virome field remain, but let us not repeat history and let bacteria steal the limelight. Viruses, fungi, and other commensals within the human microorganism ecosystem are likely equally important; we just need to overcome a few more hurdles to realize their full potential.

## Data Availability

Not applicable.

## Abbreviations

DNA: Deoxyribonucleic acid
RNA: Ribonucleic acid
dsDNA: Double-stranded DNA
ssDNA: Single-stranded DNA
NCBI: National Center for Biotechnology Information
HIV: Human immunodeficiency virus
MDA-5: Melanoma differentiation-associated protein *5*
